# Null Mutants of Individual *RABA* Genes Impact the Proportion of Different Cell Wall Components in Stem Tissue of *Arabidopsis thaliana*

**DOI:** 10.1371/journal.pone.0075724

**Published:** 2013-10-04

**Authors:** Daniel Lunn, Sanyasi R. Gaddipati, Gregory A. Tucker, Grantley W. Lycett

**Affiliations:** School of Biosciences, University of Nottingham, Sutton Bonington Campus, Loughborough, United Kingdom; Instituto de Biología Molecular y Celular de Plantas, Spain

## Abstract

In *Arabidopsis,* and other plants, the RABA GTPases (orthologous to the Rab11a of mammals) have expanded in number and diversity and have been shown to belong to eight sub clades, some of which have been implicated in controlling vesicles that traffic cell wall polymers and enzymes that synthesise or modify them to the cell wall. In order to investigate this, we have investigated whether T-DNA insertion knockouts of individual *RABA* genes belonging to different sub clades, impact on the composition of the plant cell wall. Single gene knockouts of the *RABA1*, *RABA2* and *RABA4* sub clades primarily affected the percentage composition of pectin, cellulose and hemicellulose within the cell wall, respectively, despite having no obvious phenotype in the whole plant. We hypothesise that vesicles carrying specific types of cargoes from the Golgi to the cell surface may be regulated by particular sub types of RABA proteins, a finding that could have wider implications for how trafficking systems work and could be a useful tool in cell wall research and other fields of plant biology.

## Introduction

The cell wall contains four main components, cellulose, hemicellulose, pectin and lignin with various minor contributions from proteins and inorganic compounds [1] However, the complexity within these constituents is magnified greatly by specific polysaccharide backbone and side chain linkages. In *Arabidopsis* these major components are primarily pectin with two major domains homogalacturonan and rhamnogalacturonan [2] and xyloglucan and xylan hemicelluloses [3] in the primary and secondary cell walls, respectively. In addition, lignins provide a wide array of structures through different monolignols [4]. *De novo* synthesis of the three polysaccharide components occurs in the Golgi, for pectin and hemicellulose, while cellulose synthesis occurs at the plasma membrane. Thus, pectin and hemicellulose are directly transported through the trans-Golgi network (TGN) [3], [5], while CESA proteins, involved in cellulose synthesis are cargoed to the plasma membrane, where cellulose synthesis occurs [6].

Compartmentalisation in the cells of all organisms requires tight control and organisation. Spatial localisation of many macromolecules is controlled by RAB GTPases, which have been shown to regulate vesicle traffic to many compartments within the cell through their action as molecular switches [7]. In comparison with mammalian systems, *Arabidopsis* lacks some classes of RAB proteins, but others, most notably the RABA clade (orthologous to the Rab11a of mammals) has expanded in number, diversity and perhaps roles [8], [9] and various members of the RABA clade have been implicated in trafficking to the cell wall [10].

In *Arabidopsis thaliana* there are currently 57 defined *RAB* genes. These genes are split into 8 clades which are further split into sub clades of varying size dependent on the clade. The *RABA* clade, in particular, shows a large expansion compared to the *Rab11* genes of mammalian systems. The compartmental target of most of these clades is known, mainly through localisation work [11], [12]. However, little is known about the exact role of individual RAB proteins, with redundancy often used to explain the apparent lack of visible phenotype of single gene knockouts in such studies. Since the first suggestion that a RABA1 orthologue might regulate trafficking to the cell wall [13] there has been mounting evidence that this is so for the RABA1, RABA2, RABA3 and RABA4 sub clades in plants [14]–[19] and recently it has been shown that two sub clades in *Arabidopsis*, RABA2 and RABA3 are associated with cell plate formation [20]. However, it has not been clear whether vesicles carrying different cargoes destined for the plasma membrane and the apoplast are regulated by different RABs. Here we have looked at whether individual mutations in *RAB* genes can affect the chemical composition of the plant cell wall. We have shown that mutations in different sub clades of *RABA* genes affected the cell wall composition in different ways and we suggest possible roles for the different RABA sub-clades.

## Materials and Methods

### Plant Growth

*Arabidopsis thaliana* Col-1 and mutant lines were grown under glass in the summer. Glasshouse conditions were; 22°C with 16 hour light and 8 hour dark period, light intensity of 150 µmol m^−2^s^−1^. Fifty plants of each line were placed in a randomised block structure. Stem material from the fifty plants was pooled at the senescent stage for analysis. Plants were grown in three successive months and treated as triplicates.

### Target Gene Identification

Candidate genes were selected using a screen of publically available data through Genevestigator. Plants with T-DNA knockouts of each of the genes with any expression above the cut off of 0.2 in stem were then obtained through the Nottingham *Arabidopsis* Stock Centre (NASC) services.

### Knockout Confirmation

To test for lack of expression of the target genes, RNA from fresh expanding stem tissue was extracted using RNeasy Mini Kit (Qiagen) and the complementary strand formed by incubation at 70°C for 5 minutes. The sample was then incubated at 37°C for 60 minutes, with M-MLV reverse transcriptase (Promega). PCR was then conducted on the complementary strand with specific primers for the transcripts. Lines were then tested for mRNA expression using PCR. AMV 5× reaction buffer (10 µl), dNTP 10 mM (1 µl), upstream and downstream primer (1 µl), MgSO_4_ (2 µl), AMV reverse transcriptase (1 µl), Tfl DNA polymerase (1 µl), RNA template (1 µl) as reaction mixture. Each PCR cycle comprised denaturation at 94°C for 30 seconds, annealing at 56°C for 1 minute and extension at 72°C for 1 minute. Following 30 cycles a final extension of 72°C for 5 minutes was programmed.

### Fourier Transformed Infrared Spectroscopy (FT-IR)

Dry, senescent *Arabidopsis* stem samples were ball milled (particle size 700 microns) and placed in a Bruker, Tensor 2700 IR spectrophotometer, with 80cN-m torque applied to the sampler. Samples were read in triplicate with 128 FT-IR scans per replicate. Data from background readings of 128 scans were also captured. Data were collected through the OPUS software. The data were then normalised using vector normalisation and base line corrected using the rubber band method.

PCA was conducted using data from the FT-IR in the region of 1200 nm–800 nm which is defined as the fingerprint region for sugar composition. Statistical analysis was conducted using Minitab, multivariate analysis program.

### Acetone Insoluble Solid (AIS) Production

Milled *Arabidopsis* stem (particle size 700 microns) (5 g), was weighed out and homogenised using pestle and mortar in 80% acetone (500 ml). The homogenate was then filtered through miracloth and washed using 80% acetone (500 ml) followed by 500 ml 100% acetone. The solid residue was then dried in a vacuum desiccator with phosphorus pentoxide.

### Cell Wall Analysis

Pectin was extracted from 500 mg AIS by incubating in 120 ml 50 mM Na-1,2-cyclohexylenedinitrilo-tetraacetic acid (CDTA) pH 6, for 6 hours at room temperature. The liquid fraction was removed and the residue treated with 120 ml 50 mM Na_2_CO_3_ overnight at 2°C, after which the second liquid fraction was removed. Both liquid fractions, containing the ionically and covalently bound pectin fractions, respectively were then subjected to an uronic acid assay [21]. The remaining residue was fractionated into cellulose and hemicellulose rich fractions using 4 M KOH (10 ml), for 6 h at room temperature. The residue was washed to neutral pH with H_2_O and dried to give the cellulose rich fraction. The KOH extracted material was adjusted to pH 5.5 using acetic acid and precipitated with 80% acetone. Precipitated material was collected by centrifugation at 16,500 g for 10 minutes. The pellet was then washed with ethanol twice before repeating the centrifugation step and then dried to give the hemicellulose rich fraction. Recovery of each of these fractions was assessed gravimetrically.

### Monomeric Composition

The hemicellulose rich or cellulose rich fraction (30 mg) was subjected to a two stage acid hydrolysis. 12 M sulphuric acid (1 ml) was added and the sample incubated for 1 hour at 37°C then diluted with 11 ml dH_2_O and incubated for a further 2 hours at 100°C. The sugar monomer content of the supernatant was determined by high-performance anion exchange chromatography with pulsed amperometric detection (HPAEC-PAD) (Dionex, UK) using a CarboPac PA20 column with a 50 mM NaOH isocratic system and flow rate of 0.5 ml/min at 30°C. Glucose, xylose, and galactose were used as standards with mannitol as internal standard.

### Statistics

All statistics were conducted using GenStat 14^th^ Edition, using standard ANOVA analysis to confirm significant difference between the means. In all cases the data were transformed using a log_10_ method, to allow data to fit the assumptions of the statistical test. A post-hoc Tukey test was used to determine significance between knockout lines and wildtype. In cases where the transformation was not adequate to the test, a t-test was used between wild-type and the knockout line.

### Phenotypic Characterisation of *rabA* Knockouts

For phenotypic analysis, 3 replicates were grown on separate occasions with 10 plants comprising each replicate. These 10 plants were grown with a randomised block structure under conditions of 22°C with a 16 hour light and 8 hour dark period with light intensity of 150 µmol m^−2^s^−1^. Measurements taken were based on the methods described by Boyes *et* *al*., [22].

## Results

The initial challenge in undertaking this study was to establish a set of single *rabA* gene knockouts for the genes expressed in stem tissue. To ascertain the pattern of *RAB* gene expression in stem tissue, the microarray analysis tool Genevestigator was selected, with the data of Zeef and Brown [23]. From these data, *RAB* genes shown as being expressed in stem tissue at any level above 0.2, were selected for study. The genes chosen on the basis of microarray data were *RABA1a, RABA1b, RABA1c, RABA1d, RABA1f, RABA1g, RABA1h* and *RABA1i*, the entire *RABA2* sub clade, *RABA3* and finally, *RABA4a*, *RABA4b* and *RABA4e*. Lines with T-DNA knockouts in these genes (Table S1) were obtained from the Nottingham Arabidopsis Stock Centre (NASC) [24]. Unfortunately, knockouts were not (at the time of study) available for *RABA1f, RABA1g* and *RABA1h*. Putative insert lines for *RABA1b*, *RABA2a* and *RABA2c*, on analysis, did not produce the expected insert band. From the remaining stocks, homozygous true breeding lines were isolated and the absence of gene transcripts was then confirmed by RT-PCR (Table S2, Figures S1–S4). A robust phenotypic analysis, designed to highlight minor changes, was then conducted. From these experiments no major or minor morphological differences were observed.

**Figure 1:**
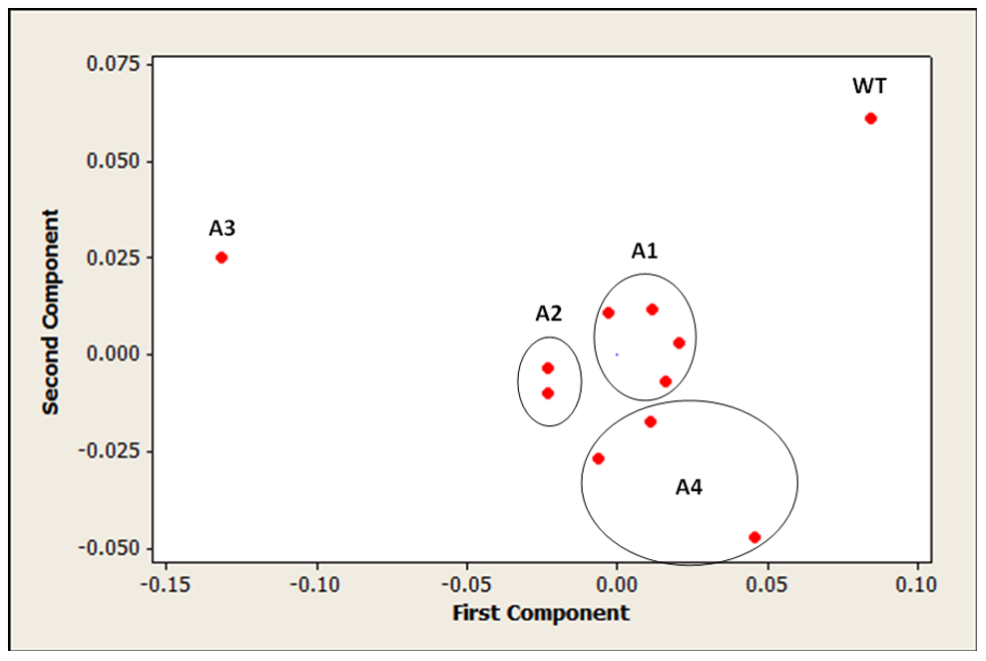
Principal component analysis (PCA) of FT-IR spectra of *Arabidopsis* stem material. Powdered stem tissue from wild type and *rabA* sub clade mutant lines of *Arabidopsis* were subjected to analysis using FT-IR. The spectral data in the region of1200 nm–800 nm was used to generate the PCA. The total number of principle components identified was 5, PC1 = 0.59 PC2 = 0.39, with the remaining components each totalling less than 0.1 of the variation.

In order to investigate possible changes in the cell wall induced by the T-DNA knockouts, we chose to analyse fully senescent tissues, to avoid the possibility that changes were due to slight differences in the stage of development of the stems. Fourier transformed infrared spectroscopy (FT-IR) was used as an initial, rapid means to assess cell wall composition. FT-IR has been used to assess cell wall composition for several years [25] and, more recently; this has been reinforced with the identification of fingerprint regions for cell wall constituents [26]. However, the spectra produced have proved difficult to resolve clearly due to the complication of analysing non-fractionated samples. Because of this, the data obtained from FT-IR were used to produce a PCA plot (Figure 1). The data show the grouping of each of the *rabA* knockout lines as compared to the wild type. The *rabA1*, *rabA2* and *rabA4* mutant lines could be seen to form non-overlapping groups which, along with the single *rabA3* mutant, were located away from the wild type.

**Figure 2:**
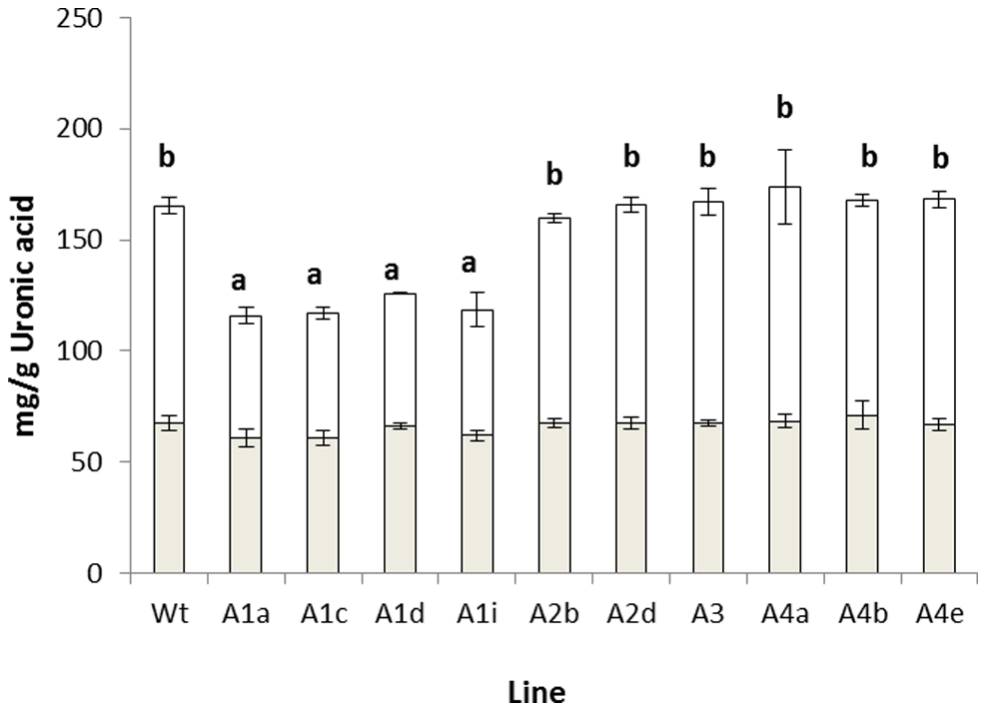
Pectin levels (as estimated by uronic acid content) in stem tissue from wild type and *rabA* knockout lines. Acetone insoluble solids were sequentially extracted with CDTA and Na_2_CO_3_ to generate ionically (clear) and covalently (grey) bound pectin, respectively represented to show individual levels and additive total pectin. Significance values are as follows; (p<0.001 (d.f,32 v.r,39.13) with letters annotating significant difference between means.

**Figure 3:**
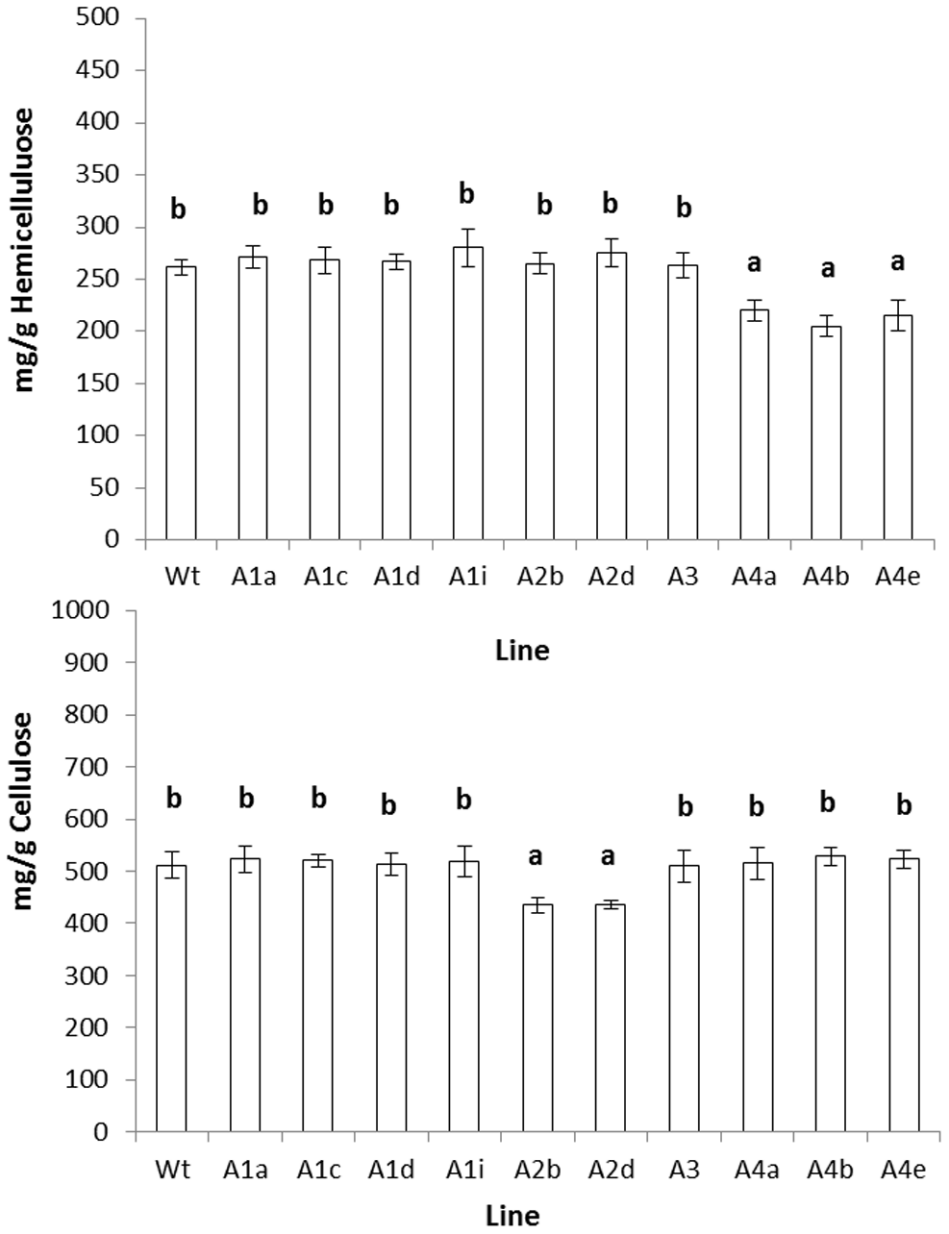
Levels of Cellulose rich and hemicellulose rich fractions in stem tissue of wild type and *rab*A knockout lines. The residue from the pectin extraction was fractionated using KOH into (A) The cellulose rich fraction and (B) The hemicellulose rich fraction. Each fraction was dried and weighed. The statistical values are as follows; “cellulose rich” fraction p<0.001 (d.f,32 v.r,6.8); “hemicellulose rich” fraction p<0.001 (d.f,32 v.r,15.04) with letters annotating significant difference between means.

In order to assess whether differences seen in the PCA may have arisen from differences in cell wall polymer composition, the proportions of cellulose, hemicelluloses and pectin were assessed through fractionation. Senescent dry stem tissue was milled to a homogeneous state and the tissue was then fractionated using CDTA and NaCO_3_ to give ionically and covalently bound pectin fractions, respectively. The data are shown in Figure 2. Pectin was estimated as uronic acid content and levels of total uronic acid (ionic plus covalently bound) were significantly reduced, in comparison to the wild type, in the knockout lines for all four members of the RABA1 sub-clade studied. Levels of covalently bound uronic acid, in contrast, were found to be similar in all lines, with the variance in total uronic acid being due to modifications in the ionically bound fraction. The residue, following pectin extraction, was subjected to treatment with 4 M KOH in order to extract the hemicellulosic fraction. The resultant hemicellulose rich and cellulose rich fractions were both quantified gravimetrically. The results of this fractionation are displayed in Figure 3. These data show significant reduction in the mass of the cellulose rich fraction obtained from both *rabA2b* and *rabA2d* mutants compared to the wild type. There was also a significant reduction in the mass of the hemicellulose rich fraction obtained from all three *rabA4* lines compared to the wild type. Mass balances were calculated for the three fractions analysed for each plant line and recoveries were found to be between 88% and 95% of the total AIS.

**Figure 4:**
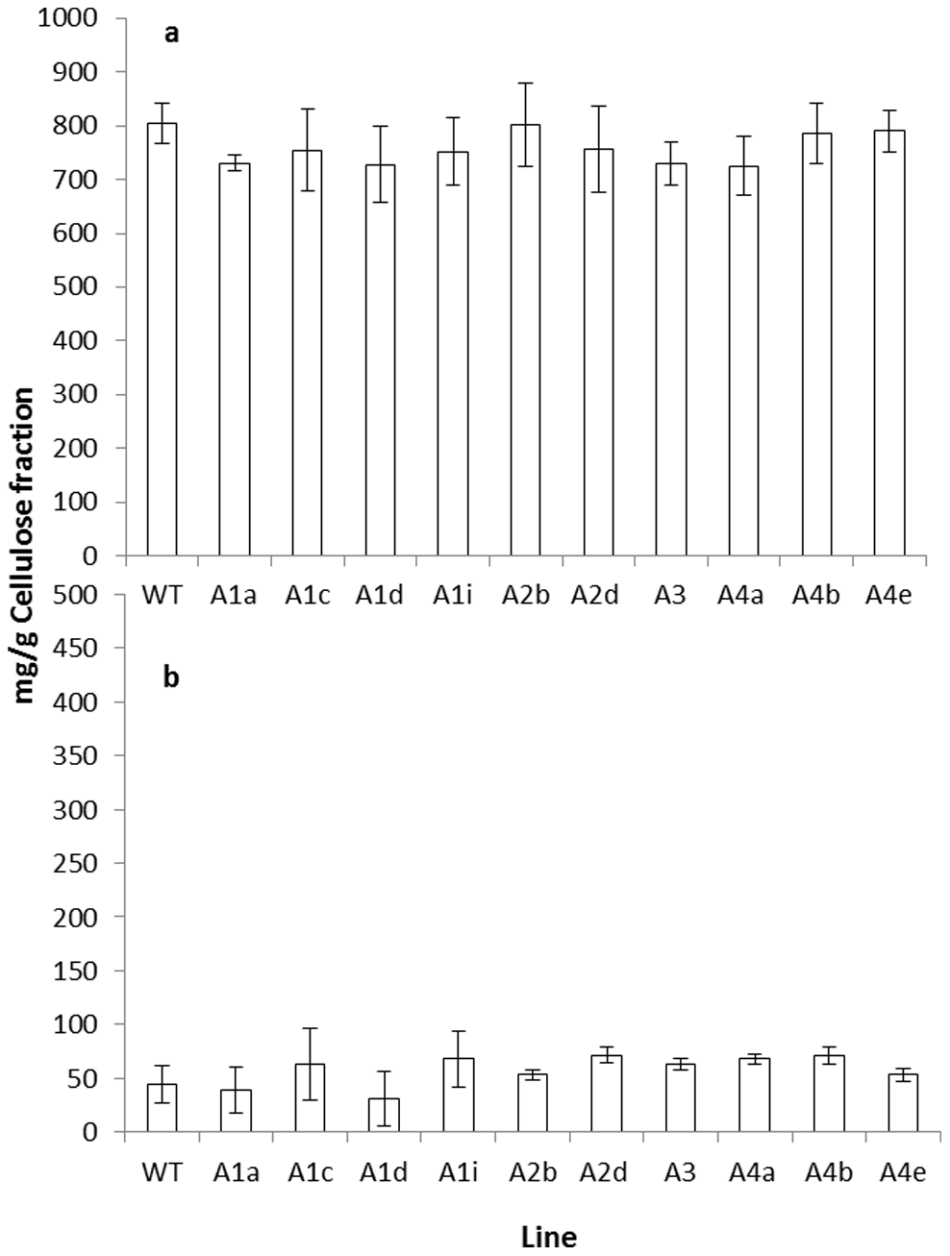
Sugar composition of the cellulose rich fraction of wild type and *rabA* knockout lines. The cellulose rich fraction was subjected to hydrolysis and monomer sugar composition in terms of (A) Glucose or (B) Xylose assessed by HPAEC. Statistical values are as follows; glucose p = 0.036 (d.f,32 v.r, 2.49); xylose p = 0.115 (d.f,32 v.r,1.82).

**Figure 5:**
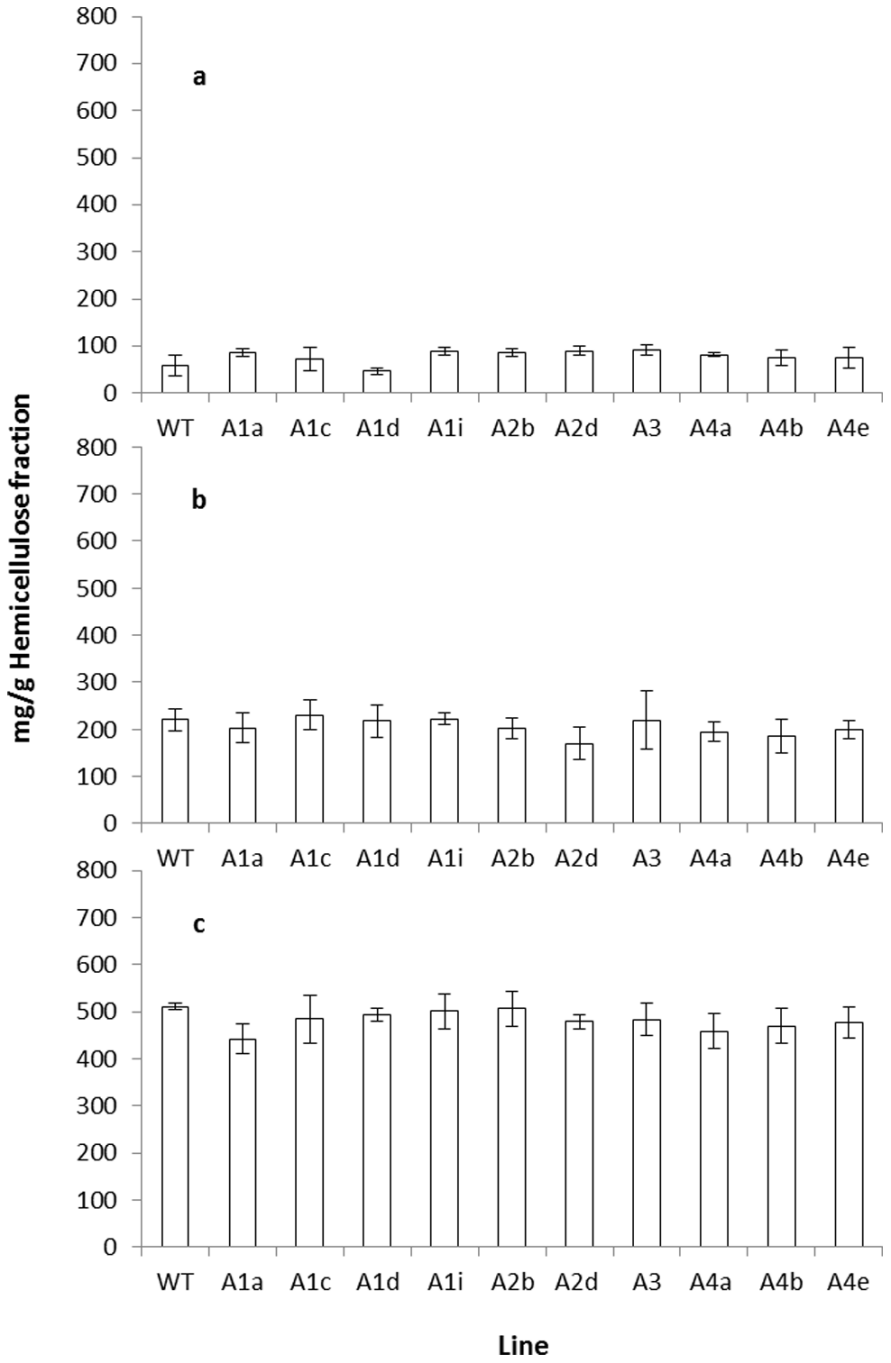
Sugar composition of the hemicellulose rich fraction of wild type and *rabA* knockout lines. The hemicellulose rich fraction was subjected to hydrolysis and monomer sugar composition in terms of (A) Galactose, (B) Glucose and (C) Xylose assessed by HPAEC. Statistical values are as follows; glucose p = 0.0494 (d.f,32 v.r,0.97); xylose p = 0.362 (d.f,32 v.r,1.17); galactose p = 0.022 (d.f,32 v.r,2.7).

The hemicellulose rich and cellulose rich fractions were further characterised for monomer sugar composition by high performance anion exchange chromatography (HPAEC) following Seaman hydrolysis. The results for the cellulose rich fraction are shown in Figure 4. As expected, glucose represents the major sugar present. The observed levels of xylose however, indicate that this “cellulose rich fraction”, again as expected, probably contains a minor amount of residual hemicellulosic material. This profile is very similar to that previously reported for the cellulosic residue from *Arabidopsis* stems [27]. In terms of glucose content the gene knockout lines affected in the production of all of the RABA proteins have similar levels to the wild type. While this would initially seem to conflict with the gravimetric results, these data show the purity of the cellulose rich fraction and the absence of an increase in residual hemicellulose in the *rabA4* knockout lines gives confidence that the patterns observed from the gravimetric analysis are not due to variances in the efficiency of the fractionation technique. The monomer composition for the hemicellulose rich fraction is shown in Figure 5. In the wild type, xylose was the predominant sugar followed by glucose and a smaller amount of galactose. The wild type thus shows a sugar profile similar to that previously described for the non-cellulosic cell wall material from *Arabidopsis* stems [28], however the relative proportions of these three sugars is slightly different in each case. Xylose was slightly more predominant in the previous study and this may reflect the different stage of development or analytical methods employed in this study. This profile was constant across all the *rabA* knockout lines. This includes the three *rabA4* knockout lines that demonstrated a reduction in the total recovery of the hemicellulose rich fraction, the proportion of xylose in the hemicellulosic rich fraction from these three knockouts being similar to the control. Again this is not in conflict with the results observed in Figure 3. These results merely suggest that *rabA4* knockouts have reduced the amount of hemicellulose and have not specifically impacted the composition, in terms of sugar content, of the hemicellulose within the cell wall.

The monomeric composition of the hemicellulose and cellulose rich fractions, along with their respective recoveries from the fractionation of the AIS were used to calculate the sugar composition of the hemicellulose and cellulose rich fractions, with respect to the AIS. These data are presented in table 1. The pattern for the cellulose rich fraction is similar to that described in figure 3 with a reduction in glucose found in the *rabA2* knockout lines. These data were supported by t-test analysis with both *rabA2b* and *rabA2d* having a value of p<0.05. The pattern for the hemicellulosic “rich” fraction is also similar to that shown in figure 4 with a reduction in both glucose and xylose in the *rabA4* knockouts as compared to wild type, again these data were supported by t-test with p values less than 0.05 with respect to glucose levels and p<0.01 with respect to xylose.

**Table 1:** Sugar composition of the “hemicellulose rich” and “cellulose rich” fractions expressed as mg/g AIS.

“Hemicellulose rich” fraction											
Knockoutline	WT	A1a	A1c	A1d	A1i	A2b	A2d	A3	A4a	A4b	A4e
Glucose	57.7±6.1	54.9±8.6	61.6±8.4	58.0±9.0	62.2±3.2	53.5±5.9	46.6±9.6	57.8±16.1	42.8±4.3	37.9±7.2	42.6±4.3
Xylose	133±1.5	120±8.8	130.0±13.8	131.8±3.6	140.1±10.4	134. 1±9.7	131.8±4.2	127.3±9.0	100.9±8.0	96.2±7.5	102.7±7.0
Galactose	15.5±5.6	23.4±2.4	19.4±6.8	12.4±1.8	25.2±2.2	23.0±2.0	24.6±2.7	24.3±2.8	18.0±1.1	15.5±3.3	16.2±4.7
**“Cellulose rich” fraction**											
**Knockout** **line**	**WT**	**A1a**	**A1c**	**A1d**	**A1i**	**A2b**	**A2d**	**A3**	**A4a**	**A4b**	**A4e**
Glucose	411.8±19.0	382.3±7.9	392.4±39.9	373.3±36.3	389.7±32.7	348.5±33.9	330.2±34.7	382.5±20.4	373.1±28.1	414.9±29.3	413.6±20.4
Xylose	22.5±8.8	20.7±11.2	32.9±17.5	15.8±13.0	35.3±13.5	23.1±1.8	31.3±3.2	32.1±2.5	35.1±2.3	37.3±4.2	27.8±3.2

## Discussion

Here we have demonstrated that *rab* single gene knockouts can affect cell wall composition. The similar result obtained for independent knockouts in several related genes rules out the possibility that these results may be due to genetic effects of the insertions themselves or to random mutations within the lines. However, it is interesting that each individual knockout gives a significant effect, which implies that these RAB proteins are not completely redundant but each RAB protein may have an independent role. This is also consistent with the finding that in a *rabA4a* mutant, a EYFP-RABA4b construct could not fully rescue the wild type pollen tube phenotype [18].

It has been shown that RABA2 and RABA3 proteins localise to the cell plate [20] and from this finding it was reasonable to assume that these RAB proteins were involved in cell wall deposition; however their exact role was not established. Here, using a different approach, namely the quantitative assessment of polymer content in mutants, we have presented evidence that supports the role of RABA2 proteins in cell wall metabolism and shows for the first time that RABA1, and RABA4 proteins also have independent roles in this process. The results indicate that *rabA1* mutations affect pectin composition, which is consistent with the finding that pectin content is reduced in fruit of a transgenic tomato line in which a *RABA* orthologue has been silenced [19]. The results also indicate that *rabA2* mutations affect cellulose composition and *rabA4* mutations affect hemicellulose composition. Hemicellulose and pectin are made in the Golgi apparatus and transported to the cell wall [3], [5]. In the case of cellulose, biosynthetic machinery is transported to the plasma membrane and cellulose is synthesised there [6].

There are various ways in which a disruption of vesicle trafficking could affect cell wall composition. CESA proteins have been found in several post-Golgi compartments and their trafficking is complex, possibly involving recycling events [29], [30]. Similarly, pectic polysaccharides and xyloglucans may be remobilised out of the cell wall after deposition and modification [31]–[33]. Therefore, the first possibility is that recycling has been affected in our mutants, though it is difficult to see how inhibition of mobilisation from the wall could cause a reduction in the content of a particular polymer. Trafficking of cell wall remodelling enzymes has also been shown be affected by inhibition of a tomato *RABA1a* orthologue [14] so a change in enzymic modification of the wall is a second possible mechanism. Finally, the most direct and obvious mechanism is that trafficking of new polysaccharides to the apoplast and new CESA complexes to the plasma membrane (PM) may have been inhibited. Further work will be needed to distinguish these possible mechanisms but, whatever the mechanism, the differential effects of mutations in members of different sub clades implies that they are regulating vesicles that carry different cargoes, whether those cargoes be newly synthesised polysaccharides, recycled polymers or enzymes.

Material trafficked to the PM and the apoplast has been thought to be carried by a default bulk flow pathway [34] and in support of this, a collection of vesicles named a mobile secretory cluster has been observed moving from the *trans*-Golgi network to the PM and the cell plate in several plant species [35], however, there is mounting evidence that there may be multiple routes from the Golgi/TGN to the PM and the cell wall. A low concentration of brefeldin A only slightly inhibited incorporation of labelled proteins into the PM and the cell wall, whereas polysaccharides were greatly affected [36]. Conversely, interference with trafficking by means of a dominant negative mutant of the syntaxin SYP121/PEN1 reduced trafficking of secreted proteins by half but did not affect incorporation of matrix polysaccharides or cellulose synthase complexes [37]. A more recent study of two inhibitors of pectin hydrolases [38], showed that PMEI1 is transported with the aid of a GPI anchor but PEIP2 is not and neither of these is affected by a SYP121 dominant mutant, whereas secreted GFP is affected.

The role of RABA GTPases in regulating different routes has been less fully investigated, though it has been shown that tomato Rab11a, a close orthologue of RABA1a is probably involved in the pathway mediated by SYP122 and not in the one mediated by SYP121 [17]. There is also very little previous evidence to say whether different cell wall polymers travel in different vesicles, though there is one microscopic study using specific antibodies that indicates that different cell wall polymers may exit the *trans-*Golgi cisternae or the *trans-*Golgi network in different vesicles [39]. Confirmation of this will be important because of the problems in ensuring that antibody binding is specific. Despite the mounting evidence that there are different routes to the cell wall and that different cargoes may travel by different routes, we believe that our data are the first to suggest that different RAB GTPases may regulate trafficking of different cargoes.

Finally, the ability to generate plants with altered cell wall composition, which have no other obvious phenotype, may also offer a useful tool for future cell wall research.

## Supporting Information

Figure S1 **Confirmation of gene transcript absence by RT-PCR analysis of** ***rabA1*** **T-DNA knockout lines.** Expected size fragments for each reaction are shown in table S2. Panel A = *rabA1a,* Panel B = *rabA1c,* Panel C = *rabA1d,* Panel D = *rabA1i.* Key to lanes: M, Marker; 1, wild type *RAB* gene expression; 2, No RT; 3, *RAB* gene expression in insert line; 4, Actin expression in insert line.
(TIF)

Figure S2 **Confirmation of gene transcript absence by RT-PCR analysis of** ***rabA2*** **T-DNA knockout lines.** Expected size fragments for each reaction are shown in table S2. Panel A = *rabA2b,* Panel B = *rabA2d.* Key to lanes: M, Marker; 1, wild type *RAB* gene expression; 2, No RT; 3, *RAB* gene expression in insert line; 4, Actin expression in insert line.
(TIF)

Figure S3 **Confirmation of gene transcript absence by RT-PCR analysis of** ***rabA3*** **T-DNA knockout line.** Expected size fragments for each reaction are shown in table S2. Key to lanes: M, Marker; 1, wild type *RAB* gene expression; 2, No RT; 3, *RAB* gene expression in insert line; 4, Actin expression in insert line.
(TIF)

Figure S4 **Confirmation of gene transcript absence by RT-PCR analysis of** ***rabA2*** **T-DNA knockout lines.** Expected size fragments for each reaction are shown in table S2. Panel A = *rabA4a,* Panel B = *rabA4b* Panel C = *rabA4e.* Key to lanes: M, Marker; 1, wild type *RAB* gene expression; 2, No RT; 3, *RAB* gene expression in insert line; 4, Actin expression in insert line.
(TIF)

Table S1 **Details of NASC stock lines.**
(TIF)

Table S2 **Details of RT-PCR primers.**
(TIF)
