# Supplementary figures and images for: Null Mutants of Individual RABA Genes Impact the Proportion of Different Cell Wall Components in Stem Tissue of Arabidopsis thaliana
Source: PLoS One. 2013 Oct 4;8(10):e75724. doi: 10.1371/journal.pone.0075724 (PMC3790814; doi:10.1371/journal.pone.0075724)

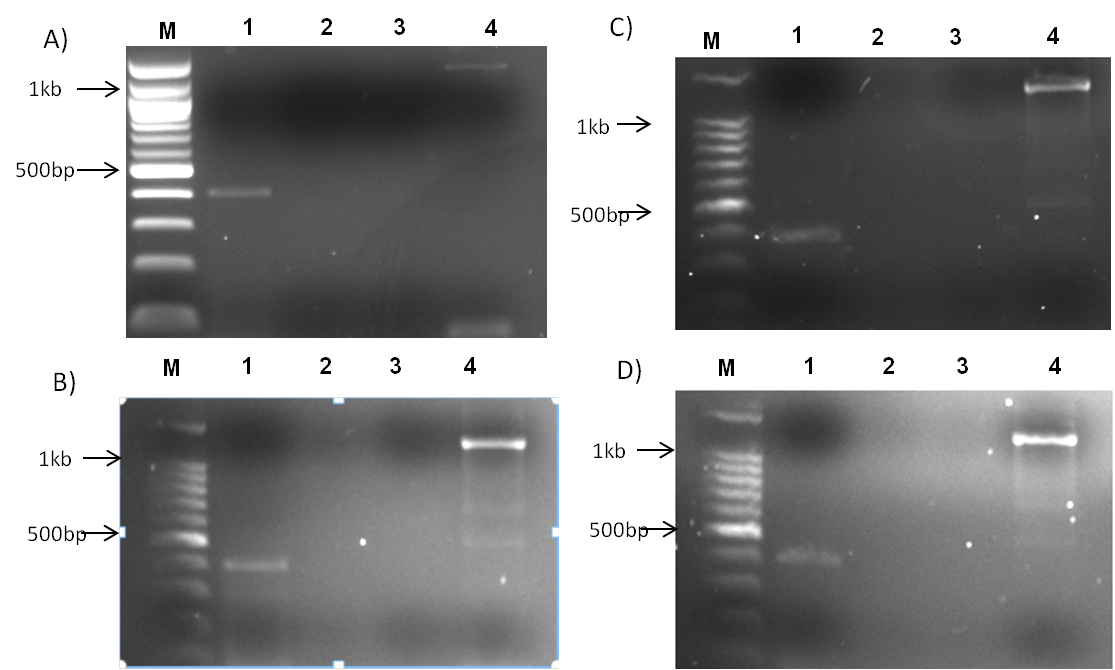

Supplement: Figure S1 — Confirmation of gene transcript absence by RT-PCR analysis of rabA1 T-DNA knockout lines. Expected size fragments for each reaction are shown in table S2. Panel A = rabA1a, Panel B = rabA1c, Panel C = rabA1d, Panel D = rabA1i. Key to lanes: M, Marker; 1, wild type RAB gene expression; 2, No RT; 3, RAB gene expression in insert line; 4, Actin expression in insert line. (TIF) [file pone.0075724.s001.tif]

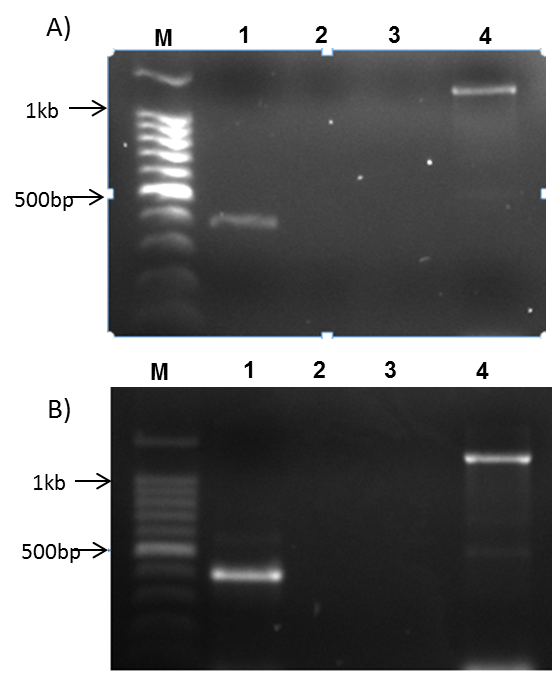

Supplement: Figure S2 — Confirmation of gene transcript absence by RT-PCR analysis of rabA2 T-DNA knockout lines. Expected size fragments for each reaction are shown in table S2. Panel A = rabA2b, Panel B = rabA2d. Key to lanes: M, Marker; 1, wild type RAB gene expression; 2, No RT; 3, RAB gene expression in insert line; 4, Actin expression in insert line. (TIF) [file pone.0075724.s002.tif]

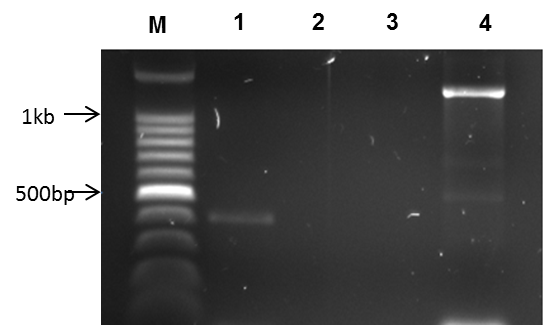

Supplement: Figure S3 — Confirmation of gene transcript absence by RT-PCR analysis of rabA3 T-DNA knockout line. Expected size fragments for each reaction are shown in table S2. Key to lanes: M, Marker; 1, wild type RAB gene expression; 2, No RT; 3, RAB gene expression in insert line; 4, Actin expression in insert line. (TIF) [file pone.0075724.s003.tif]

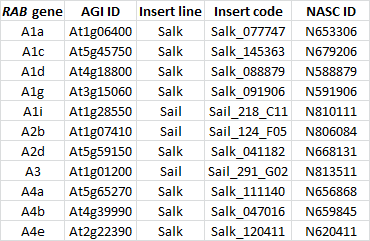

Supplement: Table S1 — Details of NASC stock lines. (TIF) [file pone.0075724.s005.tif]

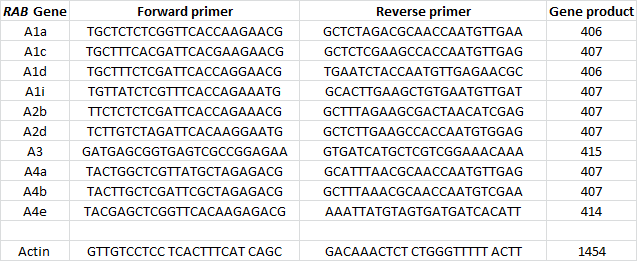

Supplement: Table S2 — Details of RT-PCR primers. (TIF) [file pone.0075724.s006.tif]
